# University of New York—Medical Department

**Published:** 1850-04

**Authors:** John W. Draper

**Affiliations:** Secretary of the Medical Faculty, 380 Fourth street, New York


UNIVERSITY OF NEW YORK.—MEDICAL DEPARTMENT.

The Medical Faculty of the University of New York regret to announce that their colleague, Professor, S. H. Dickson, whose health, previously delicate, has suffered from change of climate, has resigned the chair of the Institutes and Practice of Medicine.

In accordance with the regulations of the University, notice is hereby given, that applications will be received for the vacant professorship, addressed to the undersigned.

All applications will be regarded as confidential, and must be sent in on or before the first day of May.

JOHN W. DRAPER, M. D.,
Secretary of the Medical Faculty,
380 Fourth street, New York.

